# Protective Role of Deoxyschizandrin and Schisantherin A against Myocardial Ischemia–Reperfusion Injury in Rats

**DOI:** 10.1371/journal.pone.0061590

**Published:** 2013-04-19

**Authors:** Ruimiao Chang, Yong Li, Xingxin Yang, Yuan Yue, Lili Dou, Yanwei Wang, Weifang Zhang, Xiaoni Li

**Affiliations:** 1 College of Pharmacy, Shanxi Medical University, Taiyuan, Shanxi Province, China; 2 Department of Physiology, Shanxi Medical University, Taiyuan, Shanxi Province, China; Thomas Jefferson University, United States of America

## Abstract

**Background:**

Our previous studies suggested that deoxyschizandrin (DSD) and schisantherin A (STA) may have cardioprotective effects, but information in this regard is lacking. Therefore, we explored the protective role of DSD and STA in myocardial ischemia–reperfusion (I/R) injury.

**Methodology/Principal Findings:**

Anesthetized male rats were treated once with DSD and STA (each 40 µmol/kg) through the tail vein after 45 min of ischemia, followed by 2-h reperfusion. Cardiac function, infarct size, biochemical markers, histopathology and apoptosis were measured and mRNA expression of gp91*^phox^* in myocardial tissue assessed by RT-PCR. Neonatal rat cardiomyocytes were pretreated with DSD and STA and then damaged by H_2_O_2_. Cell apoptosis was tested by a flow cytometric assay. Compared with the I/R group: (i) DSD and STA could significantly reduce the abnormalities of LVSP, LVEDP, ±d*p*/dt_max_ and arrhythmias, thereby showing their protective roles in cardiac function; (ii) DSD and STA could significantly attenuate the infarct size and MDA release while increasing SOD activity, suggesting a role in reducing myocardial injury; (iii) tissue morphology and myocardial textual analysis revealed that DSD and STA mitigated changes in myocardial histopathology; (iv) DSD and STA decreased apoptosis (33.56±2.58% to 10.28±2.80% and 10.98±1.99%, respectively) and caspase-3 activity in the myocardium (0.62±0.02 OD/mg to 0.38±0.02 OD/mg and 0.32±0.02 OD/mg, respectively), showing their protective effects upon cardiomyocytes; and (v) DSD and STA had similar protective effects on I/R injury as those seen with the positive control metoprolol. *In vitro*, DSD and STA could significantly decrease the apoptosis of neonatal cardiomyocytes.

**Conclusions/Significance:**

These data suggest that DSD and STA can protect against myocardial I/R injury. The underlining mechanism may be related to their role in inhibiting cardiomyocyte apoptosis.

## Introduction

Acute myocardial infarction is due to the death of myocardial cells caused by thrombotic occlusion of the coronary artery. Acute myocardial infarction is the leading cause of death in human cardiovascular disease. Swift restoration of the normal blood supply is the only effective way to minimize cardiac injury. However, reperfusion itself can also cause myocardial injury and cardiac dysfunction, and is known as “reperfusion injury” [1]–[3]. Hence, mitigating myocardial ischemia–reperfusion (I/R) injury is an important means for the treatment of ischemic heart disease [4].

Fructus Schisandrae, the fruit of *Schisandra chinensis* (Turcz.) Baill. has been used as a tonic and astringent in traditional Chinese medicine (TCM) for centuries [5]. A recent study demonstrated that Fructus Schisandrae was the anti-oxidant component herb in a Chinese medicinal formula called “Sheng Mai San” which is commonly used for the treatment of coronary heart disease [6]. Investigations revealed that lignans from Schisandra, including deoxyshisandrin (DSD) and schisantherin A (STA), are the principal active constituents of Fructus Schisandrae, and that they have liver-protective, anti-tumor and anti-oxidant activities [7].

In our previous study, we found that DSD and STA could bind to rat cardiomyocyte membranes. These membranes contain many types of important receptors related to regulation of the physiological functions of heart. Hence, this finding suggested their potential role in protecting myocardial cells. The myocardial-protection activity of these compounds was then validated preliminarily by pharmacological experiments *in vivo* [8]. However, no definite evidence in this regard is available. Therefore, in the present study, we developed a new strategy to further ascertain if DSD and STA have definite protective effects on the cardiomyocyte in *in vivo* and *in vitro* experiments.

## Materials and Methods

### Ethical approval of the study protocol

The experimental procedures were conducted in adherence to the Guide for the Care and Use of Laboratory Animals published by the US National Institutes of Health (NIH, publication number 85–23, revised 1996, http://grants.nih.gov/grants/olaw/olaw.htm), and approved by the Animal Care and Use Committee of Shanxi Medical University (permit number 2009–0001). All efforts were made to minimize the number of the animals used and their suffering.

### Drugs and reagents

**Figure 1:**
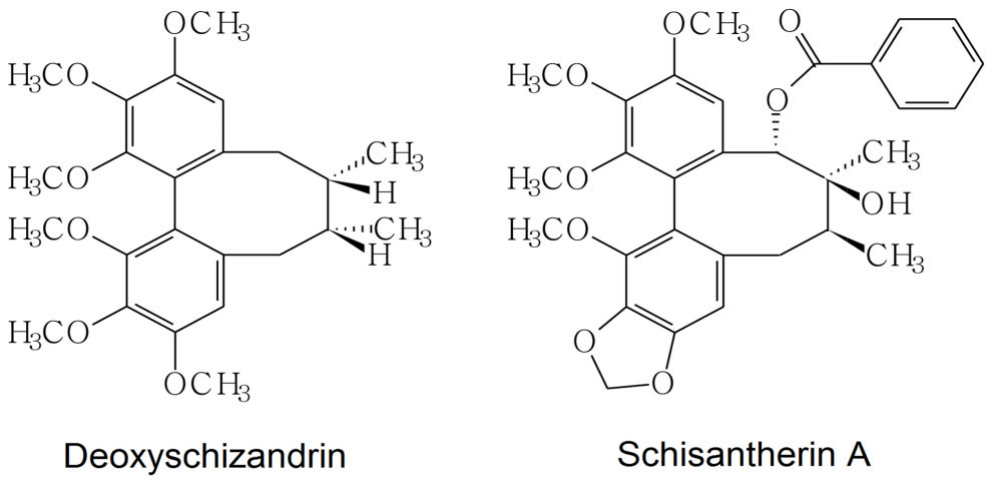
Chemical structures of DSD and STA.

DSD (Figure 1; molecular weight (MW), 267.0) and STA (Figure 1; MW 259.0) were obtained from the National Institutes for Food and Drug Control (Beijing, China). The dry powders of DSD and STA were prepared into microemulsion injections by Shanxi Yabao Pharmaceutical Group Co., Ltd. (Taiyuan, China). Metoprolol injections were supplied by ShangDong East San Lu Pharmaceutical Co., Ltd. (Sishui, China). Extreme care was taken while preparing all solutions to avoid decomposition.

### Animals

Male Wistar rats (230±10 g) supplied by the Research Animal Center of Shanxi Medical University (Shanxi, China) were used in the study. The rats were maintained under standard conditions (ambient temperature 21–23°C; with a 12-h dark–light cycle) with *ad libitum* access to food and tap water. The animals were fasted overnight before experimentation.

### Myocardial ischemia and reperfusion

According to references and a preliminary study [9], [10], a model was constructed by occluding the left coronary artery for 45 min followed by 2 h of reperfusion. This model could closely simulate acute myocardial I/R injury. In brief, rats were anesthetized with 1 g/kg urethane. The left side of the heart was exposed through a fourth intercostal thoracotomy. The heart was exposed by opening the pericardium. A 6–0 silk suture slipknot was placed at the distal third of the left anterior descending coronary artery. After 45 min of ischemia, the slipknot was released, and the myocardium reperfused for 2 h. Successful myocardial ischemia was confirmed by ST segment elevation in electrocardiographic alteration, as well as visual assessment of regional cyanosis of the ischemic region in the left ventricle (LV). Reperfusion was confirmed by ST segment reversal and a color change in the ventricular surface from cyanosis to hyperemia [11].

All animals were assigned randomly to one of five groups: (1) sham: animals underwent surgical procedures without coronary occlusion; (2) I/R: rats were pretreated with placebo (microemulsion injection without DSD or STA) at 5 mL/kg; (3) DSD: rats were subjected to DSD microemulsion injection at 40 µmol/kg; (4) STA: rats were treated with STA microemulsion injection at 40 µmol/kg; (5) metoprolol: rats were given a metoprolol injection at 2.5 mg/kg. All drugs were administered via injection into the tail vein during the early stage of reperfusion.

### Hemodynamics

Left ventricular function was monitored continuously using a Mikro-Tip® Catheter Pressure Transducer (BL420F-Powerlab, Taimeng Technology Co., Ltd., Chengdu, China). A filled catheter (0.9% NaCl with 5 IU/mL heparin) was inserted into the left ventricular cavity via the right common carotid artery. The indicators determined were left ventricular systolic pressure (LVSP), left ventricular end-diastolic pressure (LVEDP) and the maximal rates of increase and decrease in LV pressure (±d*p*/dt_max_).

### Electrocardiographic monitoring

Twelve-lead electrocardiography (ECG) was used continuously. Arrhythmias were analyzed in accordance with the criteria of the Lambeth Conventions [12]. Ventricular tachycardia (VT) was defined as a run of ≥4 consecutive ventricular premature beats. Ventricular fibrillation (VF) was defined as a ventricular rhythm without a recognizable QRS complex in which signal morphology changed from cycle to cycle, and for which it was not possible to estimate the heart rate (HR) [13]. We measured the prevalence and total duration of VT and VF during the reperfusion period.

An arrhythmia score was used to indicate the prevalence and duration of arrhythmias, i.e., 0, no arrhythmias; 1, <10 s VT or other arrhythmias, no VF; 2, 11–30 s VT or other arrhythmias, no VF; 3, 31–90 s VT or other arrhythmias, no VF; 4, 91–180 s VT or other arrhythmias, and/or <10 s reversible VF; 5, >180 s VT or other arrhythmias, and/or >10 s reversible VF; 6, irreversible VF [13]. No rat received anti-arrhythmic agents before and during occlusion and reperfusion.

### Quantification of infarct size

Infarct size was assessed with Evans Blue (Sigma–Aldrich, St. Louis, MO, USA) and triphenyltetrazolium chloride (TTC; Amresco, Solon, OH, USA) staining. At the end of 120-min reperfusion, the coronary artery was immediately re-ligated, and 2% Evans Blue solution administered intravenously to stain the normally perfused region blue and outline the area at risk (AAR). After the atria and right ventricle were excised, the LV was washed with 0.9% (physiological) saline and stored at −70°C. Frozen hearts were cut into 5–6 transverse slices (thickness, ≈2 mm) across the long axis, stained with 1% TTC (pH 7.4, 37°C) for 15 min, and then fixed in 10% formalin for 24 h [14], [15]. In the trial operation, the AAR was separated from the non-ischemic zone and incubated to differentiate the necrotic (pale) from non-necrotic (red) AAR. The area of necrosis (An), AAR, and LV were analyzed using Image Pro Plus 7.0 (Media Cybernetics, Silver Spring, MD, USA). The AAR was indicated as a percentage of the LV (AAR/LV), and the An as a percentage of the AAR (An/AAR, infarct size) [15]. The criteria for inclusion were that the AAR was >15% and <45% of the LV area [16].

### Measurement of the serum level of malondialdehyde (MDA) and superoxide dismutase (SOD) activity

In all groups, before sacrificing the animal at the end of 2 h of reperfusion, a blood sample (4 mL) was collected from the right common carotid artery and placed into non-anticoagulant tubes. After leaving at room temperature for 2 h, samples were centrifuged for 15 min (3,000× *g*, 4°C). Aliquots of the supernatant were removed and stored at −70°C until assay. According to the manufacturer's instructions, serum levels of MDA and SOD activity were measured by colorimetric analyses using a spectrophotometer with the relevant detection kits (Nanjing Jiancheng Bioengineering Institute, Nanjing, China).

### RNA isolation and analysis of gp91*^phox^* mRNA by Real-time reverse transcription-polymerase chain reaction (RT-PCR)

Total RNA was extracted from the heart using Trizol Reagent (Invitrogen, Carlsbad, CA, USA) according to manufacturer instructions. RT-PCR for rat gp91*^phox^* was undertaken as described previously [17], [18]. The primer for gp91*^phox^* was: 5′ ACGCCCTTTGCCTCCATTCT 3′ (sense) and 5′ GCTTCAGGGCCACACAGGAA 3′ (antisense) (accession number U43384).

### Histopathology

At the end of reperfusion, similar myocardial tissues were excised immediately, rinsed using ice-cold 0.9% saline and fixed in 10% formalin solution (pH 7.4) for 48 h. Thereafter, the tissues were dehydrated and embedded in paraffin wax. The paraffin-embedded tissues were sectioned (4-µm thick), stained with hematoxylin and eosin (H&E) and studied by light microscopy. Comparisons of anatomical landmarks assured that similar sections were taken from all animals.

### Image analyses

From each of seven cases per group, ≥5 representative images were archived. The images from the same anterior left ventricular wall under 400-fold magnification were saved as 32-bit files without compression or image processing (i.e., normalization or equalization). In these images, similar tissue orientation was achieved by placing the long axis of the myocardial fibers parallel to the horizontal frame axis. Only images without non-muscular elements (i.e., coronary arteries, epicardial fat, endocardial structures and blank fields) were captured and evaluated. To assess textural uniformity, files were converted to 8-bit grey and analyzed using ImageJ public-domain software (http://rsbweb.nih.gov/ij/) with Gray Level Co-occurrence Matrix (GLCM) [19]. The angular second moment (ASM) showed the uniformity of an image; contrast (CT) showed the amount of local variations in an image; correlation (CR) measured the pixel linear dependencies; the inverse difference moment (IDM) illustrated the homogeneity of an image; and entropy (ET) indicated the amount of order in an image [20].

### Terminal deoxynucleotidyl transferase dUTP nick end labeling (TUNEL) assay

The myocardial tissues embedded in paraffin were sectioned (thickness, 2 µm) for the TUNEL assay with a commercial *in situ* cell death detection kit (Roche, Mannheim, Germany). According to the manufacturer's protocol, the deoxyribonucleic acid nick ends were visualized with diaminobenzidine. After counterstaining with H&E, the apoptotic cells in these sections became visible. The number of 8-Oxo-2′-deoxyguanosine (8-OHdG)-positive nuclei and TUNEL-positive nuclei was quantified in four randomly selected fields/section by Image Pro Plus 7.0 (Media Cybernetics) and averaged. The number of TUNEL-positive cells was expressed as a percentage of the total amount of cells [21].

### Determination of caspase-3 activity

Caspase-3 activity in myocardial tissues was determined with a Caspase-3 Assay kit (KeyGen Biotech Co., Nanjing, China) according to manufacturer instructions. Briefly, tissue was homogenized in ice-cold lysis buffer by a homogenizer. Homogenates were centrifuged at 10,000× *g* for 5 min at 4°C and the supernatants collected. Then, protein concentrations were measured by the bicinchoninic acid (BCA) method (Nanjing Jiancheng Bioengineering Institute). To each well of a 96-well plate, supernatant containing 200 µg of protein was loaded and incubated with 5 µL of the caspase-3 substrate N-acethyl-Asp-Glu-Val-Asp (DEVD)-*p*-nitroanilide at 37°C for 4 h in the dark. The absorbance was then measured at 405 nm with a microplate reader. The activity of caspase-3 in tissue samples was calculated as optical density per mg protein (OD/mg) [22].

### Neonatal rat cardiac cell cultures

Cardiac cells were isolated from 1–3-day-old neonatal Wistar rats and cultured as described previously [23]. Briefly, rats were sacrificed and their hearts excised. After scalpel homogenization, heart tissue was incubated with 0.25% (*w/v*) trypsin overnight at 4°C after treatment with 0.1% (*w/v*) collagenase for 20 min at 37°C. Cardiomyocytes were enriched by centrifugation and plated in Dulbecco's modified Eagle's medium (DMEM) supplemented with 10% (*v/v*) fetal calf serum at 37°C and 5% (*v/v*) CO_2_ for 24 h. Cardiomyocytes were allowed to adhere overnight in serum-deprived medium before treatment.

### Cell viability assay

Cell viability was determined via a colorimetric method using the MTT assay. Cells at the exponential phase were seeded at 5×10^4^ cells/well in 96-well plates. After treatment with 0.1–10 µM DSD or STA for 24 h, 15 µL of 5 mg/mL MTT solution was added to each well, and wells incubated at 37°C and 5% (*v/v*) CO_2_ for 4 h. The supernatants were aspirated, the formazan crystals in each well were dissolved in 150 µL of dimethyl sulfoxide, and optical density at 570 nm read on a microplate reader.

### Drug treatment and cardiomyocyte injury induced by exposure to H_2_O_2_

To evaluate the protective effects of DSD and STA, cells were pretreated for 24 h with drugs at 1×10^6^ cells/well in six-well plates, and washed twice with D-Hank's solution before the addition of H_2_O_2_. Cardiomyocytes were then exposed to 200 µM H_2_O_2_ for 2 h [24], [25]. Control cells were also incubated under identical conditions.

### Flow cytometric analyses for apoptosis

Apoptotic cells were detected by annexin V and propidium iodide (PI) double labeling following the manufacturer's protocol as described elsewhere [23]. Briefly, cells were harvested at the indicated time periods, washed twice with cold phosphate-buffered saline (PBS), and then re-suspended in 500 µL binding buffer. This was followed by staining with 5 µL of annexin V-fluorescein isothiocyanate (FITC) and 5 µL of PI for 15 min at room temperature in the dark. Flow cytometry (BD FACSAria II) was used to assess the percentage of cell apoptosis.

### Statistical analyses

Data are the means ± SD. One-way ANOVA was used to compare groups. Arrhythmia parameters among different groups were analyzed using the chi-square test. *P*<0.05 was considered significant. Samples were analyzed in a blinded fashion.

## Results

### Effects on hemodynamics

LVSP and ±d*p*/dt_max_ in the I/R group decreased significantly (*P*<0.01 *vs* sham, *P*<0.01 *vs* baseline), whereas LVEDP increased significantly at the end of 2 h of reperfusion (*P*<0.01 *vs* sham, *P*<0.01 *vs* baseline) (Table 1). Compared with that in the I/R group, there was a significant recovery in LVSP, LVEDP and ±d*p*/dt_max_ in STA- and metoprolol-administrated groups (all *P*<0.01); DSD had more visible improvements in LVSP, LVEDP and +d*p*/dt_max_ (*P*<0.01). There were no significant change in HR between the baseline and 2-h reperfusion groups, except the metoprolol group (from 369.8±3.6 to 372.2±2.3, *P*<0.05).

**Table 1:** Effects of various administrations upon hemodynamics in a model of ischemia–reperfusion in rats.

Group	HR (beat/min)	LVSP (mmHg)	LVEDP (mmHg)	+d*p*/dt_max_ (mmHg/s)	−d*p*/dt_max_ (mmHg/s)
Sham					
Baseline	371.9±2.2	150.7±2.4	1.5±0.38	7486±142	5650±136
Reperfusion 2 h	371.7±3.1	146.3±2.8^&&^**^‡^	1.2±0.5^&&^**^†^	7396±155^&&^**	5518±175^&&^**
I/R					
Baseline	372.6±3.2	146.9±5.4	1.3±0.6	7455±124	5217±338
Reperfusion 2 h	370.4±3.5	112.4±3.4**^‡^	5.2±0.6**^‡^	4562±345**^‡^	4186±238**^‡^
Metoprolol					
Baseline	369.8±3.6	146.6±8.9	1.4±0.2	7489±157	5411±212
Reperfusion 2 h	372.2±2.3^†^	129.6±3.2^&&‡^	3.0±0.6^&&‡^	5423±315^&&‡^	4553±187^&&‡^
DSD					
Baseline	370.5±2.6	147.5±3.4	1.5±0.4	7117±553	5599±507
Reperfusion 2 h	371.0±2.6	124.5±3.8^&&^**^‡^	3.6±0.4^&&^**^‡^	4901±297^&&^**^‡^	4381±173^‡^
STA					
Baseline	371.7±2.8	147.1±3.3	1.3±0.3	7218±437	5264±212
Reperfusion 2 h	370.9±3.3	122.6±2.0^&&^**^‡^	3.5±0.4^&&^**^‡^	4781±169^&&^**^‡^	4764±257^&&^*^‡^

Data are the mean ± SD. Values marked with ^&&^ *P*<0.01 are significantly different from I/R. Values marked with * *P*<0.05 or ** *P*<0.01 are significantly different from metoprolol. Values marked with ^†^ *P*<0.05 or ^‡^ *P*<0.01 are significantly different from baseline. LVSP, LVEDP and ± d*p*/dt_max_ represent left ventricular systolic pressure, left ventricular end-diastolic pressure, and the maximal rates of increase and decrease in LV pressure, respectively.

### Effects on arrhythmias

**Figure 2:**
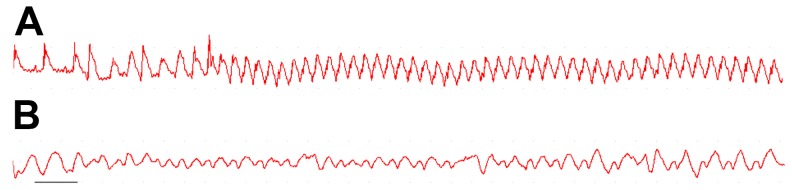
Examples of ventricular tachycardia (VT) and ventricular fibrillation (VF). A shows sustained VT of regular morphology and rate. B shows one example of terminal VF. Scale bar, 300 ms.

The mortality index in the DSD, STA and metoprolol groups was 12.5% (2/16) respectively, but there was no significant difference in all groups (*P*>0.05, Table 2). Typical ventricular tachycardia (VT) and ventricular fibrillation (VF) in the experiment are shown in Figure 2. Compared with I/R rats, the duration of VT during reperfusion in the DSD, STA and metoprolol groups was significantly reduced (*P*<0.01, Table 2), whereas there was no significant difference in the prevalence of VT between the four groups (*P*>0.05). Every drug tested caused a decrease in the prevalence and duration of VF as well as the arrhythmia score compared with the I/R group during the stabilization and reperfusion phases (*P*<0.01). When compared with each other, the arrhythmia score was lower in the metoprolol group than that in DSD- or STA-treated rats (*P*<0.01, *P*<0.05).

**Table 2:** Effects of various administrations on mortality and arrhythmias on ischemia–reperfusion-induced arrhythmias in rats.

Group	n	Mortality	Ventricular tachycardia		Ventricular fibrillation		Arrhythmia score
		(%)	Duration (s)	Prevalence (%)	Duration (s)	Prevalence (%)	
Sham	16	0 (0/16)	0^&&^	0^&&^ (0/16)	0^&&^	0^&&^ (0/16)	0^&&**^
I/R	16	25 (4/16)	140.23±71.28^**^	100 (12/12)	39.17±31.61^**^	100^**^ (12/12)	5±0^**^
Metoprolol	16	12.5 (2/16)	18.65±9.76^&&^	92.8 (13/14)	0^&&^	0^&&^ (0/14)	1.71±0.73^&&^
DSD	16	12.5 (2/16)	46.10±20.19^&&*^	92.8 (13/14)	0^&&^	0^&&^ (0/14)	2.64±0.93^&&**^
STA	16	12.5 (2/16)	62.53±34.46^&&**^	78.6 (11/14)	0.5±1.29^&&^	14.3^&&^ (2/14)	2.50±1.40^&&*^

Data are the mean ± SD. Values marked with ^&&^ *P*<0.01 are significantly different from I/R. Values marked with * *P*<0.05 or ** *P*<0.01 are significantly different from metoprolol.

### Effect on myocardial infarct size

**Figure 3:**
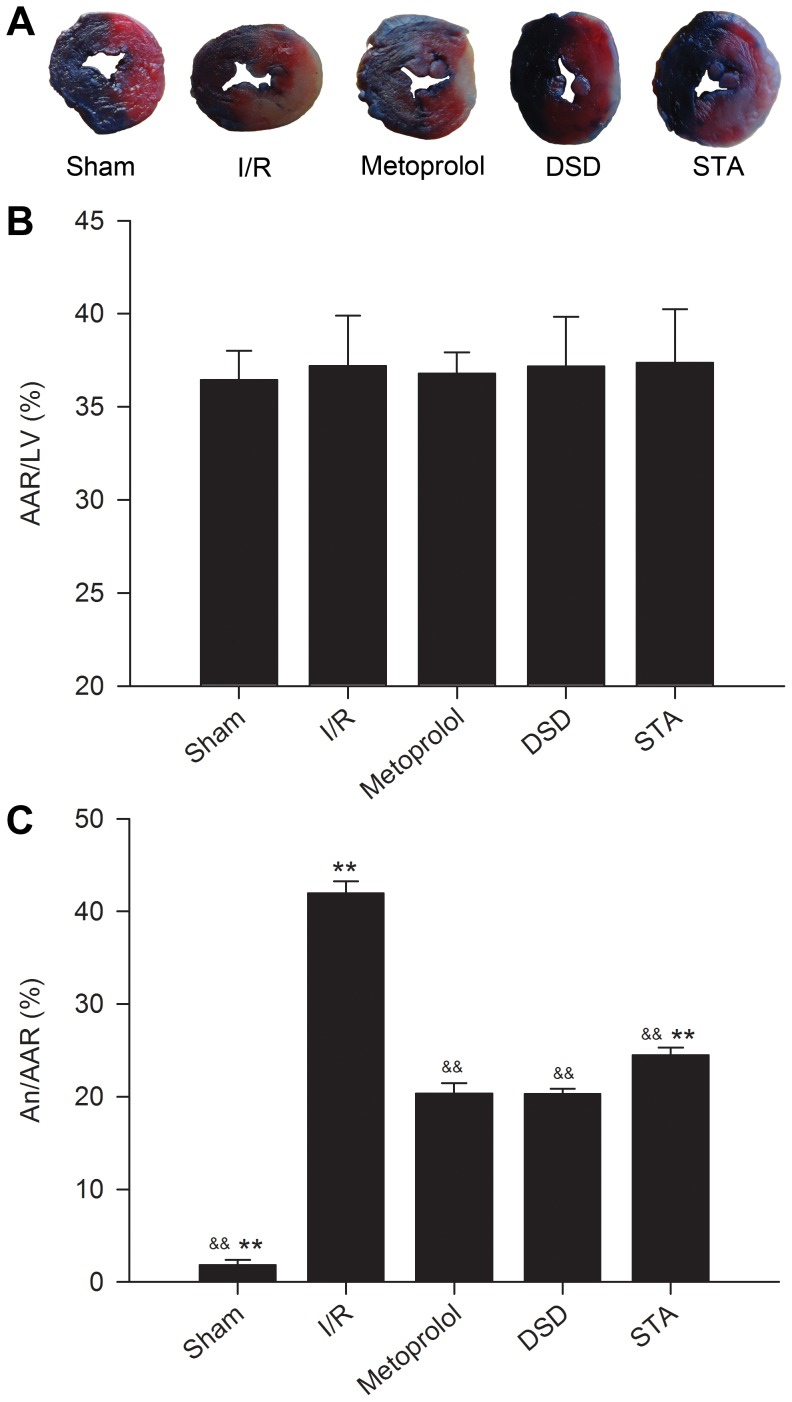
Infarct size in rats of various groups subjected to I/R injury. Representative illustrations of infarct size as stained by Evans Blue and TTC (A). Bar graphs show AAR as a percentage of the LV (B) and An as a percentage of the AAR (C). Traces of the mean values (± SD, vertical lines). ^&&^ *P*<0.01 *vs* I/R, ^**^ *P*<0.01 *vs* metoprolol.

AAR/LV was comparable among all groups (Figure 3B), averaging between 35% and 40%. This finding suggested that the ischemic area induced by coronary ligation was approximately identical among each experimental group (*P*>0.05), so these groups were comparable. An/AAR in the I/R group was significantly increased compared with that in the sham group (41.92±1.31% *vs* 1.83±0.54%, *P*<0.01, Figure 3C). The metoprolol group had a significantly smaller infarct size 20.33±1.14% (*P*<0.01 *vs* I/R). Administration of DSD and STA at the onset of reperfusion significantly reduced infarct size to 20.28±0.56% and 24.48±0.81% (*P*<0.01 *vs* I/R), respectively.

### Effect on MDA level and SOD activity

**Figure 4:**
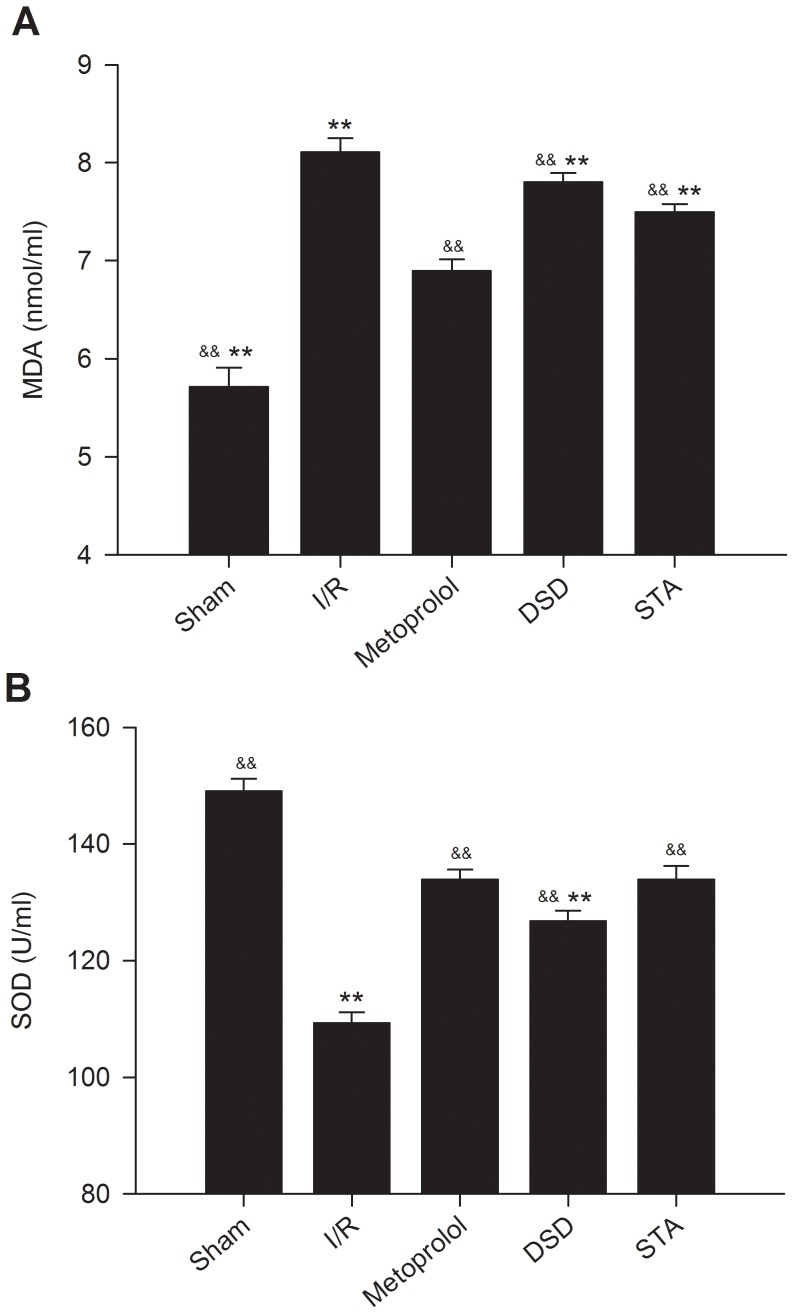
Effect on serum MDA levels and SOD activity after I/R injury in each group of rats. Serum samples were collected after 2 h of reperfusion; MDA concentrations were assayed according to the thiobarbituric acid (TBA) method (A); SOD activities were measured by the xanthine oxidase method (B). Traces of the mean values (± SD, vertical lines). ^&&^ *P*<0.01 *vs* I/R, ^**^ *P*<0.01 *vs* metoprolol.

The serum concentration of MDA was significantly higher in the I/R group compared with sham groups (*P*<0.01) (Figure 4A). In contrast, in all groups treated with DSD, STA, and metoprolol, serum MDA concentrations were lower than in the I/R group (*P*<0.01), suggesting that treatment with these drugs generated a low risk of lipid peroxidation.

In rats treated with DSD, STA, and metoprolol, the increase in serum SOD activities after 45-min ischemia and 2-h reperfusion was significantly more pronounced compared with that seen in I/R rats, confirming the anti-oxidation properties of DSD, STA, and metoprolol in rats (*P*<0.01, Figure 4B). In addition, serum SOD activity was significantly reduced in DSD groups compared with that observed in the metoprolol group (*P*<0.01).

### Gene expression of gp91*^phox^*

**Figure 5:**
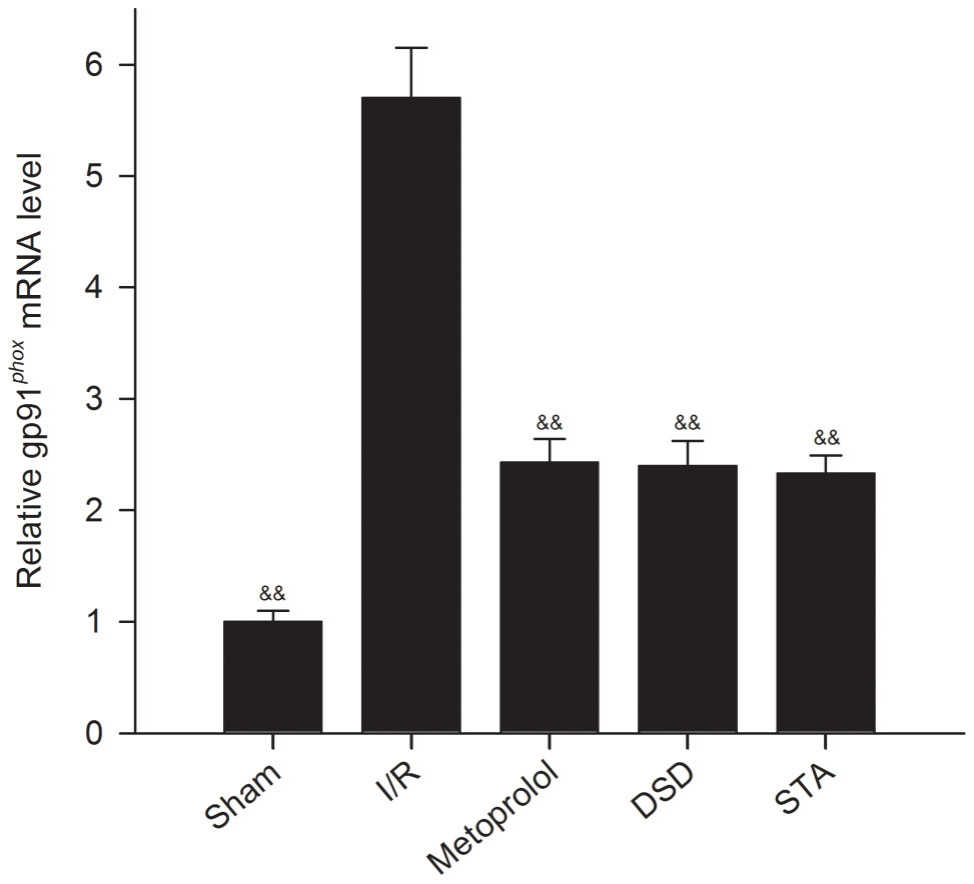
Gp91*^phox^* mRNA level was analyzed by real-time RT-PCR on extracted total RNA. Threshold cycles (Ct values) were normalized to their corresponding GAPDH mRNA and the comparative mRNA levels determined by the ΔΔCt method. Traces of the mean values (± SD, vertical lines). ^&&^ *P*<0.01 *vs* I/R.

Gene expression level of gp91*^phox^* was measured in the myocardium of rats by RT-PCR. We demonstrated that I/R leads to upregulation of expression of the NADPH oxidase subunit gp91*^phox^* (Figure 5, *P*<0.01 *vs* sham). Deficiency of DSD, STA and metoprolol significantly reduced the levels of gp91*^phox^* mRNA induced by I/R stimulation (*P*<0.01 *vs* I/R).

### Effect on histopathology

**Figure 6:**
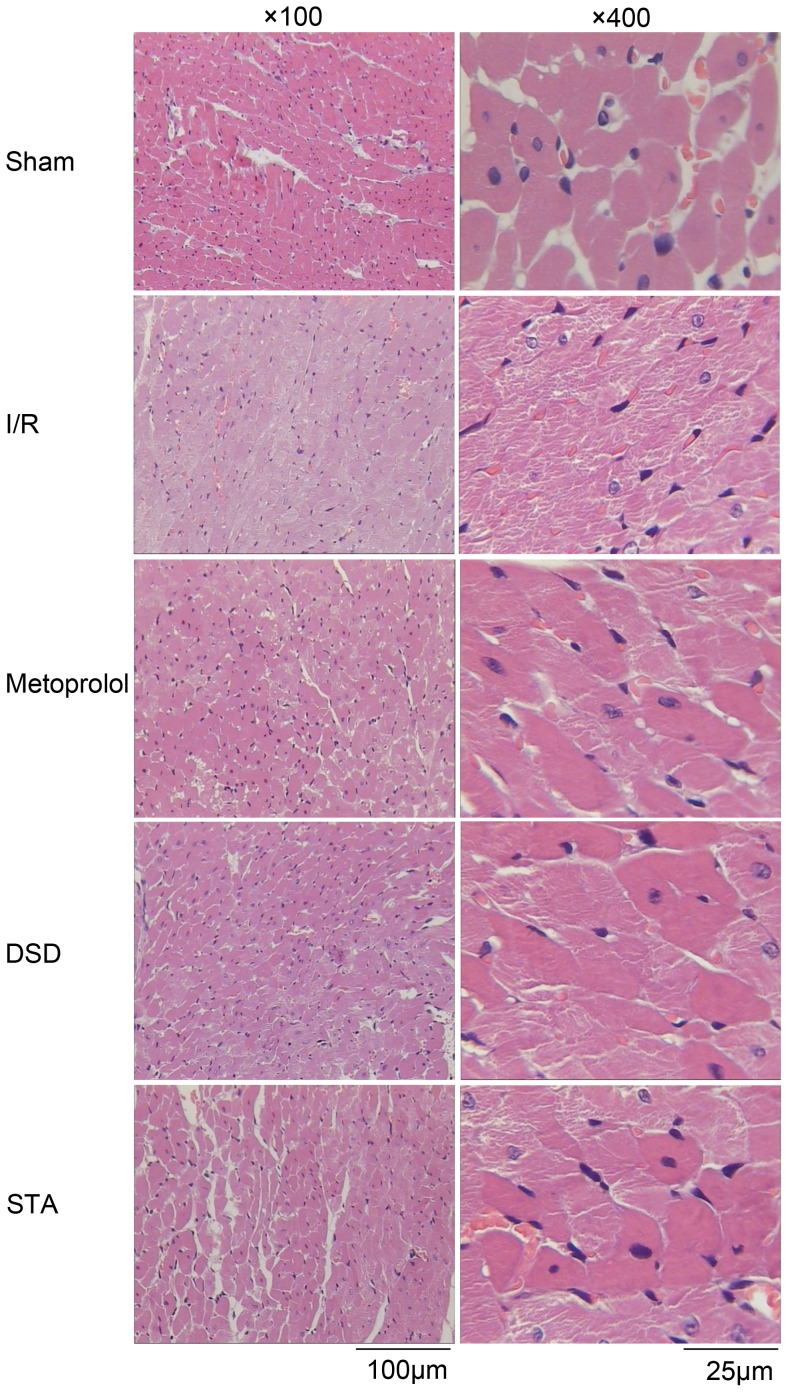
Representative slides of H&E staining. A comparison of H&E staining in myocardial tissue from sham-operated rats or rats with different pretreatments after ischemia for 45 min and reperfusion for 2 h. Representative images are shown at 100× and 400× magnifications and the scale bars represent 100 µm and 25 µm respectively. In specimen from I/R rats, focal attenuation of the sarcoplasm and focal loss of striations were observed in the ischemic region. Hyperemic blood vessels and extravasated erythrocytes were observed among the muscle fibers. Specimens from the metoprolol, DSD and STA groups showed attenuation of the sarcoplasm in the ischemic areas and general maintenance of muscle striations. Myofiber disruptions were barely noticeable. Hyperemia and extravasated erythrocytes were seen at a lesser extent as compared with I/R.

In the ischemic region of the myocardium in the I/R group, focal attenuation of the sarcoplasm, focal loss of striations, vasodilatation and congestion were observed. Virtually all the nuclei of the myocardial cells had disappeared, and myocardial fibers were so necrotic that their outlines were barely visible (Figure 6). In addition, “wavy” forms of myocardial fibers (a typical finding in ischemia) were observed in some areas. In the specimens from the DSD-, STA- or metoprolol-treated rats, attenuated sarcoplasm in the ischemic region and signs of hyperemia similar to I/R were observed. However, unlike I/R, striations were, in general, maintained.

### Effect on image texture features

Compared with the sham group, the parameters of angular second moment (ASM), contrast (CT), correlation (CR) and entropy (ET) in the I/R group had visible changes (*P*<0.01, Table 3). Compared with the I/R group, DSD and metoprolol had more visible improvements in ASM and ET (*P*<0.01 to 0.05), whereas STA had more visible improvements in ASM, CT and inverse difference moment (IDM) (*P*<0.01 to 0.05).

**Table 3:** Effects of various administrations on gray level co-occurrence matrix parameters in a model of ischemia–reperfusion in rats.

Group	ASM	CT	CR	IDM	ET
Sham	0.0851±0.0108^&&**^	0.5358±0.1533^&&*^	0.2884±0.0290^&&**^	0.5852±0.0609	2.8311±0.3423^&&**^
I/R	0.0407±0.0039^*^	0.9185±0.1141	0.1443±0.0377	0.5275±0.0415	3.5749±0.0325^**^
Metoprolol	0.0544±0.0067^&^	0.7543±0.1064	0.1575±0.0333	0.5934±0.0436	3.2509±0.01151^&&^
DSD	0.0578±0.0124^&&^	0.7474±0.1957	0.1561±0.0396	0.5720±0.0897	3.1836±0.01379^&&^
STA	0.0551±0.0070^&&^	0.6881±0.1375^&^	0.1143±0.0470	0.6127±0.0600^&^	3.3658±0.0904

Data are the mean ± SD. Values marked with ^&^ *P*<0.05 or ^&&^ *P*<0.01 are significantly different from I/R. Values marked with * *P*<0.05 or ** *P*<0.01 are significantly different from metoprolol. ASM, CT, CR, IDM and ET represent angular second moment, contrast, correlation, inverse difference moment and entropy, respectively.

### Effect on the apoptosis of myocardial cells

**Figure 7:**
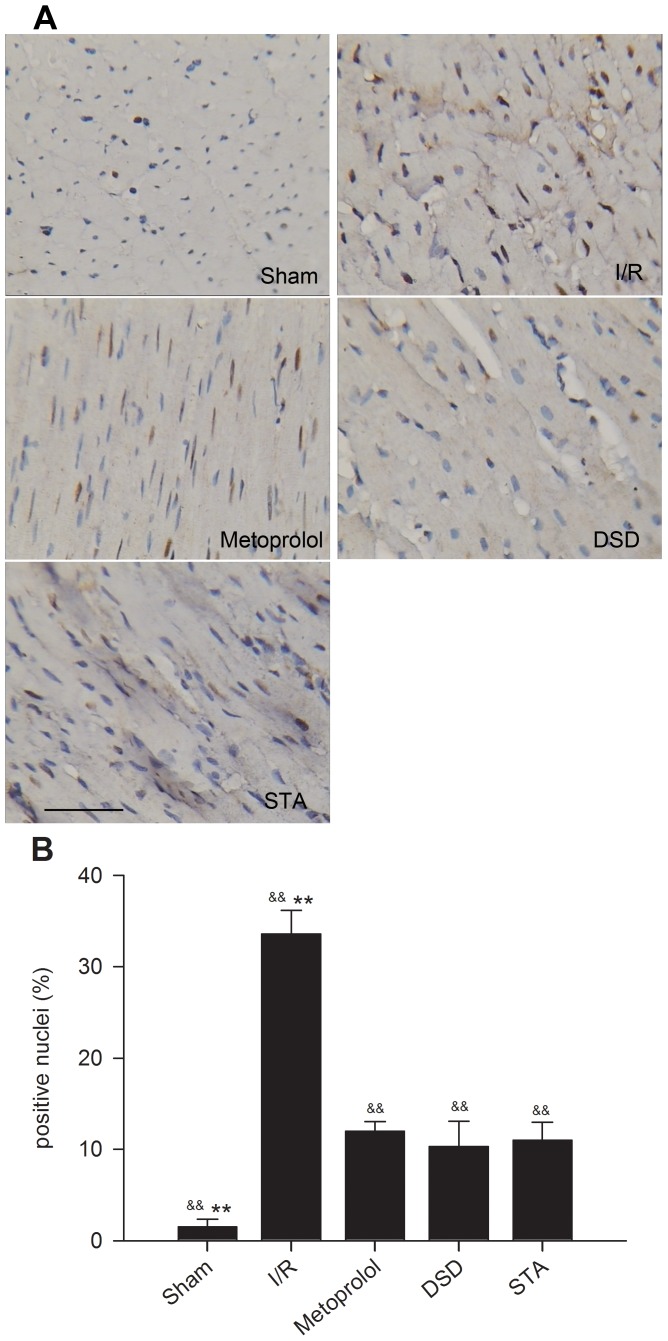
Percentages of positive nuclei in the myocardial tissues of various groups. (A) Representative photomicrographs of *in situ* detection of DNA fragments from sham-operated rats or rats with different pretreatments subjected to 45 min of ischemia followed by 2 h of reperfusion. Arrowheads indicate positive nuclei for TUNEL staining (magnification, ×250). (B) Bar graph shows the percentages of TUNEL-positive nuclei in sham, I/R and treated groups. Traces of the mean values (± SD, vertical lines). ^&&^ *P*<0.01 *vs* I/R, ^**^ *P*<0.01 *vs* metoprolol. Scale bar, 40 µm.

Two hours after reperfusion, no apoptotic cells were observed in the heart from sham-operated rats (1.50±0.84%). An increased number of TUNEL-positive nuclei in the I/R, DSD, STA and metoprolol groups was observed compared with sham rats, suggesting increased apoptosis after surgery in these animals. The marked appearance of dark brown (TUNEL-positive, Figure 7A) apoptotic cells was noted. Treatment with DSD, STA and metoprolol led to decreased percentages of positive nuclei compared with the I/R group (all *P*<0.01, Figure 7B). In contrast, there was no significant difference in the number of apoptotic cells in DSD-, STA-and metoprolol-treated rats after 2-h reperfusion (*P*>0.05).

### Effect on caspase-3 activity

**Figure 8:**
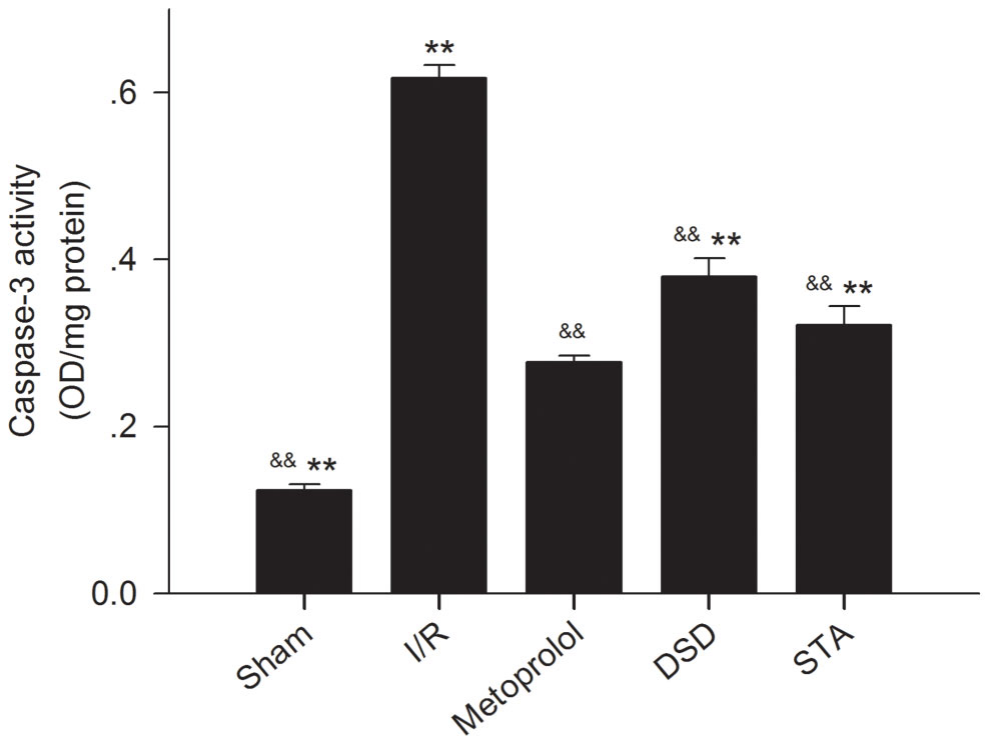
Activity of caspase-3 in the myocardial tissues of rats of various groups. Traces of the mean values (± SD, vertical lines). ^&&^ *P*<0.01 *vs* I/R, ^**^ *P*<0.01 *vs* metoprolol.

Apoptosis has a key role in the formation of infarct areas. Caspase-3 has been identified as an important protein in the final pathway of apoptosis. Therefore, we performed an assay to measure caspase-3 activity. The activity of caspase-3 in the myocardium was significantly greater in I/R group rats than in sham-group rats (I/R: 0.62±0.02 OD/mg *vs* sham: 0.12±0.01 OD/mg, *P*<0.01) (Figure 8). After delivering medication, the activity of caspase-3 induced by I/R decreased (DSD: 0.38±0.02 OD/mg, STA: 0.32±0.02 OD/mg, metoprolol 0.28±0.01 OD/mg *vs* I/R: 0.62±0.02 OD/mg, all *P*<0.01).

### Effect on apoptosis induced by H_2_O_2_

**Figure 9:**
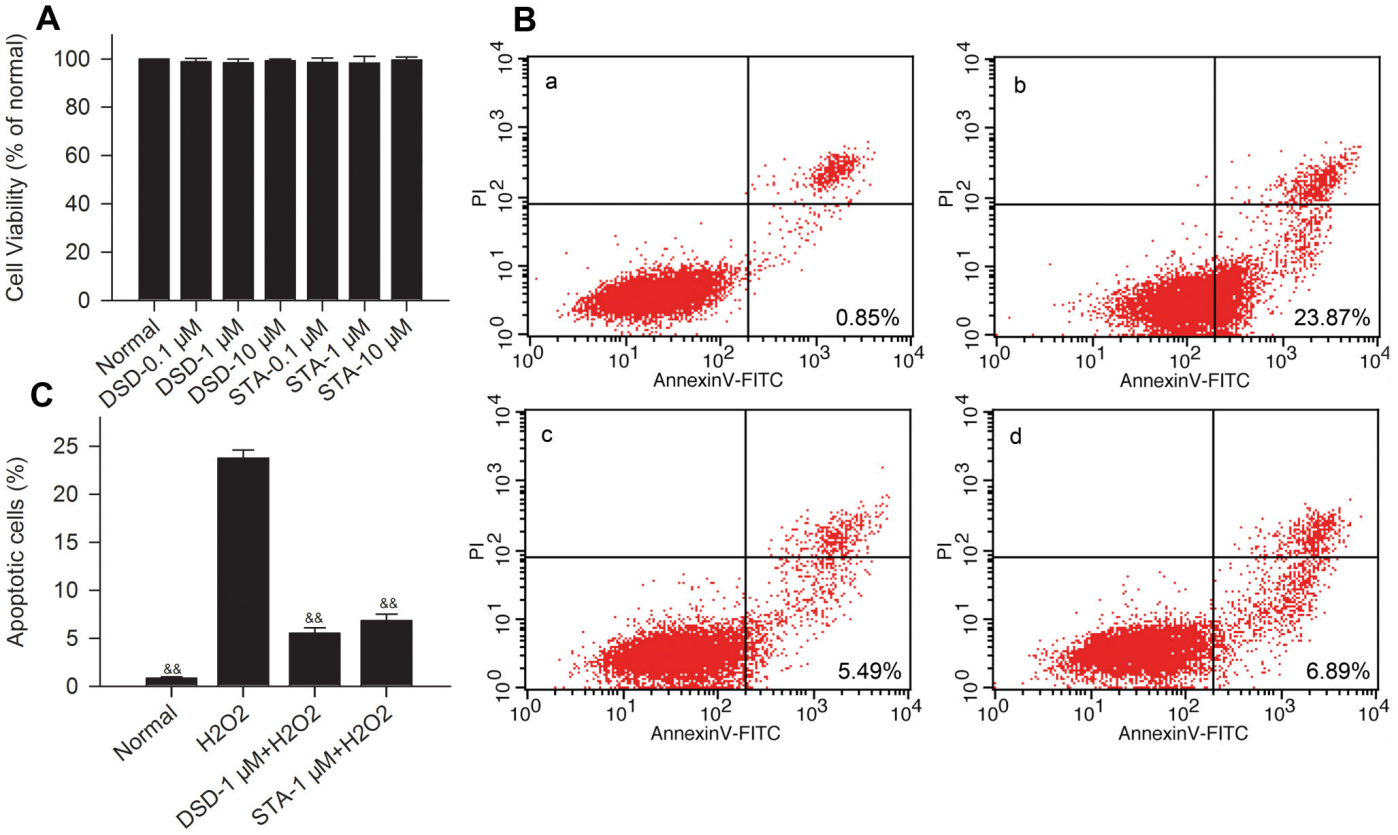
DSD and STA protect cardiomyocytes from H_2_O_2_-induced apoptosis. (A) The cytotoxicity of DSD and STA (0.1–10 µM) was measured in cardiomyocytes after 24-h incubation by MTT. (B) Different concentrations of DSD and STA (1 µM) were pretreated 24 h before incubation of cells with 200 µM H_2_O_2_ for 2 h. Normal cells (a); control cells, exposure to 200 µM H_2_O_2_ for 2 h (b); 1 µM DSD-pretreated cells, 2 h after exposure to 200 µM H_2_O_2_ (c); 1 µM STA-pretreated cells, 2 h after exposure to 200 µM H_2_O_2_ (d). (C) Statistical analysis of the flow cytometric analysis data. Data represent the means ± SD. ^&&^ *P*<0.01 *vs* H_2_O_2_.

To examine the toxicity of these drugs, cardiomyocytes were treated with DSD and STA (0.1–10 µM) and incubated for 24 h. DSD and STA did not influence viability in cultured cardiomyocytes up to 10 µM (Figure 9A). H_2_O_2_ significantly (*P*<0.01, *vs* control) increased cardiomyocyte apoptosis as determined by annexin-V staining (Figures 9B and C). However, apoptosis was significantly (*P*<0.01) reduced by pretreatment with 1 µM DSD and 1 µM STA (Figures 9B and C).

## Discussion

Although some reports have suggested the protective effects of aqueous extracts of Fructus Schisandrae on adriamycin-induced cardiotoxicity [26], studies upon the effective substances in Fructus Schisandrae have not been performed. As the main active components of Fructus Schisandrae, lignans have been shown in various studies to have diverse effects, such as pretreatment on physical exercise-induced muscle damage [27], anti-proliferative effects on tumor cells [28], [29] and anti-hepatitis B virus (HBV) activity *in vitro* [30]. This is in addition to their well-studied influence on the liver [31]–[33].

DSD and STA are the major bioactive lignans isolated from Fructus Schisandrae [34]. In our previous study, we developed an analytical method for screening the active ingredients from Fructus Schisandrae by combining cardiac muscle/cell membrane chromatography with liquid chromatography–tandem mass spectrometry [8]. Cell membrane chromatography (in which a cell membrane enriched in certain receptors is used as the stationary phase) can be applied in the analysis and screening of active ingredients of TCMS. Different types of cell membrane chromatography have been developed for various target cells and have been used for screening active components from different TCMs [35]. As noted previously, in our research we detected and identified two active components (DSD and STA) from Fructus Schisandrae. We then noted the efficacy of intravenous administration of DSD or STA in limiting infarct size in rats with acute myocardial I/R injury [8], prompting their protective action upon cardiomyocytes. However, very little information is available regarding the effects of DSD and STA on the heart. Therefore, in the present study, we ascertained if DSD or STA had any influence upon myocardial I/R damage.

Metoprolol is a selective β1-adrenoceptor antagonist. It is used extensively in clinical or pre-clinical settings for the treatment of acute myocardial infarction and heart failure [36]. For instance, administration of metoprolol has been shown to attenuate cardiac ventricular remodeling, cardiac hypertrophy, oxidative stress, and cardiomyocyte apoptosis in a model of heart failure in the rabbit [37]. Using metoprolol as the positive control, we tested if infusion of DSD or STA influenced cardiac function, myocardial infarct size, histopathology, and cardiomyocyte apoptosis. Our results demonstrated that DSD and STA exerted significant protective effects upon I/R injury that were similar to those observed with metoprolol.

Oxidative stress has been considered to play a major part in heart damage during I/R injury [38]. As the main proponents of oxidative stress, free radicals are actively involved in the I/R injury, which can be reflected by measurement of the products of free-radical attack on biological substrates (MDA) as well as intracellular and extracellular anti-oxidant capacity (SOD). MDA is one of the end products of lipid peroxidation. It can result in severe cell damage by causing polymerization and crosslinking of membrane components. Thus, MDA is an indirect marker of injury due to lipid peroxidation. SOD is the main endogenous anti-oxidant enzyme that can neutralize free radicals and protect tissues from the harmful effects of free radicals and other active oxygen species; hence SOD is an important marker of the scavenging capacity of free radicals [39]–[41]. In addition, NADPH or NADH oxidase is an inducible electron transport system found in cells that transfer reducing equivalents from NADPH or NADH to oxygen, which results in generation of the superoxide anion. The enzyme complex comprises membrane subunits (gp91*^phox^* and p22*^phox^*, which form flavocytochrome b558) and at least four cytosolic proteins (p40*^phox^*, p47*^phox^*, p67*^phox^*, and Rac1/2, which form the cytosolic complex). The membrane subunit of NADH oxidase, gp91*^phox^* (Nox2), has at least three other homologs, Nox1, Nox3, and Nox4. gp91*^phox^* and Nox4 are expressed in cardiomyocytes [42]. We found that DSD or STA could significantly decrease MDA release, gp91*^phox^* mRNA expression and increase the activity of SOD during myocardial I/R. It is therefore likely that DSD and STA can protect against myocardial I/R injury (at least in part) by attenuating oxidative myocardial damage.

It is thought that apoptosis has a key role in mediating cell death after I/R [43], [44]. Apoptosis can mediate myocardial I/R injury by three potential mechanisms. Apoptosis can: reduce the number of myocardial cells (resulting in the loss of the pumping function of the heart); suppress the conduction system in the heart (leading to arrhythmias); and initiate cardiac remodeling and pathological changes. For quantitative analyses of myocardial apoptosis, TUNEL staining is used widely to detect DNA damage. Caspases are responsible for the deliberate disassembly of the cell into apoptotic bodies during apoptosis. Cells possess multiple caspases, of which caspase-3 activity is required at the step at which a protease cascade pathway converges [45]. Hence, caspase-3 is another widely used biochemical marker for detecting apoptosis. According our *in vivo* and *in vitro* studies, we found that DSD or STA could reduce the myocardial injury induced by I/R by inhibiting apoptosis and inhibit the H_2_O_2_-induced apoptosis of neonatal rat cardiac cells.

In conclusion, the results of the present study provide novel evidence of the cardioprotective activity of DSD and STA in myocardial injury induced by I/R. Our findings give impetus for further investigation into the molecular mechanisms, clinical implications and potential role of DSD and STA as adjuvant in the therapy of cardiovascular diseases.
